# Immunoreactivity of Muscarinic Acetylcholine M2 and Serotonin 5-HT2B Receptors, Norepinephrine Transporter and Kir Channels in a Model of Epilepsy

**DOI:** 10.3390/life11040276

**Published:** 2021-03-26

**Authors:** Enes Akyuz, Zuleyha Doganyigit, Yam Nath Paudel, Betul Koklu, Emin Kaymak, Chiara Villa, Alina Arulsamy, Mohd. Farooq Shaikh, Orrin Devinsky

**Affiliations:** 1 Department of Biophysics, Faculty of International Medicine, University of Health Sciences, Istanbul 34668, Turkey; 2 Department of Histology and Embryology, Faculty of Medicine, Yozgat Bozok University, Yozgat 66100, Turkey; zuleyha.doganyigit@yobu.edu.tr (Z.D.); emin.kaymak@yobu.edu.tr (E.K.); 3 Neuropharmacology Research Strength, Jeffrey Cheah School of Medicine and Health Sciences, Monash University Malaysia, Bandar Sunway 47500, Selangor, Malaysia; yam.paudel@monash.edu (Y.N.P.); alina.arulsamy@monash.edu (A.A.); farooq.shaikh@monash.edu (M.F.S.); 4 Faculty of Medicine, Yozgat Bozok University, Yozgat 66100, Turkey; betulkoklu24@gmail.com; 5 School of Medicine and Surgery, University of Milano-Bicocca, 20900 Monza, Italy; chiara.villa@unimib.it; 6 Comprehensive Epilepsy Center, Department of Neurology, NYU Langone School of Medicine, New York, NY 10010, USA

**Keywords:** PTZ, chronic epilepsy, M2, norepinephrine, 5-HT2B

## Abstract

Epilepsy is characterized by an imbalance in neurotransmitter activity; an increased excitatory to an inhibitory activity. Acetylcholine (ACh), serotonin, and norepinephrine (NE) may modulate neural activity via several mechanisms, mainly through its receptors/transporter activity and alterations in the extracellular potassium (K^+^) concentration via K^+^ ion channels. Seizures may disrupt the regulation of inwardly rectifying K^+^ (Kir) channels and alter the receptor/transporter activity. However, there are limited data present on the immunoreactivity pattern of these neurotransmitter receptors/transporters and K^+^ channels in chronic models of epilepsy, which therefore was the aim of this study. Changes in the immunoreactivity of epileptogenesis-related neurotransmitter receptors/transporters (M2, 5-HT2B, and NE transporter) as well as Kir channels (Kir3.1 and Kir6.2) were determined in the cortex, hippocampus and medulla of adult Wistar rats by utilizing a Pentylenetetrazol (PTZ)-kindling chronic epilepsy model. Increased immunoreactivity of the NE transporter, M2, and 5-HT2B receptors was witnessed in the cortex and medulla. While the immunoreactivity of the 5-HT2B receptor was found increased in the cortex and medulla, it was decreased in the hippocampus, with no changes observed in the M2 receptor in this region. Kir3.1 and Kir6.2 staining showed increase immunoreactivity in the cerebral cortex, but channel contrasting findings in the hippocampus and medulla. Our results suggest that seizure kindling may result in significant changes in the neurotransmitter system which may contribute or propagate to future epileptogenesis, brain damage and potentially towards sudden unexpected death in epilepsy (SUDEP). Further studies on the pathogenic role of these changes in neurotransmitter receptors/transporters and K^+^ channel immunoreactivity may identify newer possible targets to treat seizures or prevent epilepsy-related comorbidities.

## 1. Introduction

Epilepsy is a debilitating disease as well as a global health concern, affecting nearly 70 million people worldwide [1]. Although there are many anti-seizure medications (ASMs) available in the current market [2] that may provide symptomatic relief for epileptic patients, about 30% of these patients still have little to no control of their devastating epileptic seizure condition [3]. Despite the rapid scientific advances in understanding the molecular biology, pathology, genetics and neurophysiology of epilepsy [4], efficient treatment strategies for drug-resistant epilepsy and prevention interventions for epilepsy-related comorbidities remain unsolved [5]. Since epilepsy results from an imbalance between the excitatory and inhibitory neurotransmitters, a deeper understanding of the neurotransmission mechanisms in epilepsy may suggest newer and more effective approaches to prevent or treat epilepsy and its associated comorbidities, as well as other neuropsychiatric disorders such as autism and schizophrenia, which all share similar imbalances in the excitatory/inhibitory ratio [6].

While glutamate and gamma-aminobutyric acid (GABA) are the main neurotransmitters governing neuronal excitability and inhibition in epilepsy, other neurotransmitters such as serotonin (5-HT), norepinephrine (NE) and acetylcholine (ACh), which also play important roles in neuronal transmission, should not be dismissed. Glutamatergic and gamma-aminobutyric acid (GABA)-ergic systems comprise a cell-to-cell specific modulatory system, while 5-HT, NE, and ACh comprise a more general (affecting general neuronal populations) modulatory system, where both modulatory systems have been implicated in modulating seizure thresholds and epilepsy pathogenesis [7,8]. Interestingly, different neurotransmission pathway dysfunctions may contribute to different epilepsy pathologies [9]. For example, the dopamine-norepinephrine-epinephrine cycle may activate the hormonal and neural pathways of excitatory and inhibitory seizure effects [10], while the serotonergic pathways may modulate the cortical tone via postsynaptic and presynaptic receptors, which may influence emotion, cognition, and motor functions directly and indirectly (e.g., ACh release) [11]. In addition, neurotransmitter receptors may also play a key role in epilepsy pathology. The antagonist of the serotonin receptor, 5HT-2B/2C, managed to increase the seizure threshold in a mouse electroshock model and protected against Pentylenetetrazol (PTZ)-induced tonic/myoclonic seizures [12]. However, in a retrospective case series, the selective 5-HT2C agonist lorcaserin suppressed motor seizures in children and young adults with treatment-resistant epilepsy [13]. These contrasting effects of the serotonin receptor suggest that downstream activity such as ion channels may have a more crucial role in epilepsy than just the neurotransmitter receptor activation/inhibition. 

Neurotransmitters regulate ion channel activities, including the inwardly rectifying K^+^ channels (Kir), an ion channel that is often associated with the epilepsy pathology [14,15]. For example, the Kir3.1 channel is activated at the muscarinic Ach receptor-2 (M2) [16] by the ACh neurotransmitter, which has been shown to maintain resting membrane potential (RMP) and reduce cellular excitability [17]. Kir3 channels and M2 receptors are also candidates for autonomic dysfunctions accompanying epilepsy leading to detrimental epilepsy comorbidities [18]. Adenosine triphosphate (ATP) sensitive K^+^ (K_ATP_) channels (Kir6), on the other hand, are normally found closed and may protect against hypoxia and ischemia conditions in the hippocampus and cortex [19], but may also regulate neuronal excitability and spontaneous neuronal firing in the striatum of epileptic conditions [20]. These latter two studies indicate that the location/area of this neurotransmission and ion channel activation in the brain may result in varying neuronal outcomes and, thus, may also exhibit varied pathology/immunoreactivity in an epilepsy model. For instance, seizures arising in the temporal-limbic regions alter brainstem centres that control cardiac and respiratory functions [21], while temporal lobe epilepsy (TLE) may cause neuronal degeneration and abnormal neurogenesis that may lead to chronically increased hippocampal associated functional loss [22]. Furthermore, knowledge regarding whether the seizures or the changes in the immunoreactivity and activity of these receptors and ion channels appear first in epilepsy conditions remains undetermined. 

Therefore, in the present study, we aimed to investigate the immunoreactivity changes of neurotransmitter receptors; M2, 5-HT2B receptors, NE transporters, and ion channels; Kir3.1 and Kir6.2, in the cortex, hippocampus, and medulla in a PTZ kindling rodent model of chronic epilepsy. The findings from this study will hopefully aid in elucidating a clearer epileptogenesis pathway via the neurotransmitter systems, especially in the chronic stages, thus advancing targeted-intervention studies for epilepsy.

## 2. Materials and Methods

Wistar albino male rats (280–380 g, *n* = 20) were obtained from Kayseri Erciyes University’s Research Center. Animals were housed in a controlled environment with a temperature of 24 ± 2 °C and humidity of 60% under a 12-h light/dark cycle. They were given free access to water and food ad libitum. All procedures were performed within the recommendations of the Guide for the Care and Use of Laboratory Animals adopted by the National Institutes of Health (NIH, Bethesda, MD, USA) and the Helsinki Declaration. The experimental protocols for this study were approved by the Animal Ethics Committee of the Kayseri Erciyes University (numbered: 19/027). All efforts were made to minimize animal suffering during each procedure. Animals were acclimatised to the room prior to any experimentation to avoid distress. 

### 2.1. PTZ-Kindling Epilepsy Model 

Epilepsy was induced in the animals through PTZ-kindling (generalized tonic-clonic seizures). First, the PTZ solution was prepared by dissolving PTZ (P6500, Sigma-Aldrich, St. Louis, MO, USA) in an isotonic saline (0.9% NaCl). Rats were randomly divided into two groups; Group 1 (*n* = 10), which is a control group that only received intraperitoneal (i.p.) injection of 0.9% saline, and Group 2 (*n* = 10), which is a PTZ-kindling group that received i.p. injection of 35 mg/kg of PTZ solution. The PTZ-kindling epilepsy model in this study was induced based on an already established protocol [23]. Briefly, animals in Group 2 were intraperitoneally injected with a sub-convulsive dose of PTZ (35 mg/kg) three times a week, on alternate weekdays. PTZ injection was continued until seizure kindling was achieved, but never longer than 4 weeks. Seizure kindling was considered achieved when the animals showed a Score 4 or 5 after three consecutive injections of PTZ [24]. 

A month after kindling, the final dose for PTZ susceptibility was performed with a single PTZ injection (50 mg/kg, i.p.). The seizure reaction after the 50 mg/kg PTZ was compared to the initial 35 mg/kg PTZ, and successful epileptogenesis in the animals was considered achieved when there was a significant enhancement in the reaction [25]. After each injection (PTZ/saline), the rats from both groups were each placed individually in a transparent Plexiglas cage and their convulsive behaviour was independently monitored for 30 min. The seizure severity, the latency of seizure onset, and the seizure duration of each animal were measured and analysed by a blinded experimenter. The seizure severity was scored using the commonly utilized Racine’s classification system:
**Score****Criteria**Score 0No responseScore 1Facial movements, ear and whisker twitchingScore 2Myoclonic convulsions without rearingScore 3 Myoclonic jerks, upright position with clonic forelimb convulsionsScore 4 Clonic-tonic convulsionsScore 5Generalized clonic-tonic seizures with loss of postural controlScore 6Death

**Figure 1:**
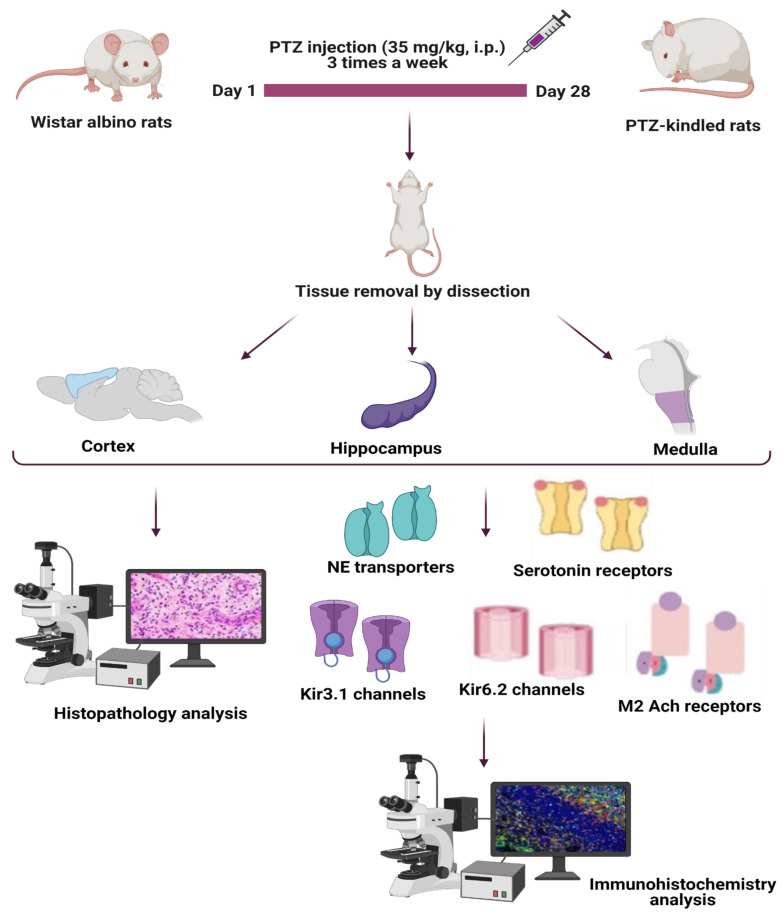
Pictorial representation depicting the current experiment. This figure was created with BioRender.com (accessed on 7 March 2021). Ach, Acetylcholine; Kir, inwardly rectifying K^+^; NE, norepinephrine; PTZ, Pentylenetetrazol

After the final seizure measurements were recorded, the animals were anesthetized with ketamine/xylazine (90/10 mg/kg, i.p.) and were sacrificed humanely. The brains of the animals were extracted for immunohistochemistry analysis (Figure 1).

### 2.2. Histological and Pathological Staining

Immunohistochemistry (IHC) staining (Figure 1) was performed as per the methodology described in the previous literature [26]. The cerebral cortex, hippocampus, and medulla tissues were dissected and immediately fixed with 4% formaldehyde solution. Then, the tissues were embedded in paraffin blocks before 5–6 μm sections of each tissue were obtained via a microtome. These sections were placed on glass slides. When ready to use, the slides were placed in an alcohol solution of ascending concentration gradient (70, 80, and 90%), each for 5 min. Then, the slides were placed in a 100% alcohol solution for 5 min and then in xylene solution for 1 min (twice). Next, Haematoxylin and Eosin (H&E) staining was performed on the tissue sections before proceeding towards visualization. The gross histopathological changes (e.g., necrosis or haemorrhage) of the tissue were evaluated under Olympus BX51 model microscope. 

As for the immunoreactivity of the M2 and 5-HT2B receptor as well as the NE transporter in the cerebral cortex, hippocampus, and medulla, this was determined using the avidin-biotin-peroxidase IHC methodology. Similarly, the 5–6 μm sections were kept on slides at 60 °C overnight. They were then deparaffinized and rehydrated by passing them through xylene and through graded alcohol series (100%, 95%, and 70%). Next, the slides were washed three times for 5 min with phosphate buffer solution (PBS). The antigen retrieval step was performed by boiling the tissue sections 5 times, 3 min each at 600 W in a microwave oven with 5% citrate buffer. The slides were then kept in the same buffer solution for 20 min. Next, the sections were washed with PBS and were treated with 3% hydrogen peroxide (H_2_O_2_) for 5 min to prevent endogenous peroxidase activity. Any non-specific binding was blocked with normal horse serum at room temperature for 20 min and washed with PBS. Primary antibodies against Kir3.1 (mouse monoclonal, 1:200, Alomone Labs, Jerusalem, Israel (APC-005)), Kir6.2 (rabbit polyclonal, 1:200, Alomone Labs, Jerusalem, Israel (APC-020)), muscarinic acetylcholine receptor M2, mAChR (rabbit polyclonal, 1:200, Alomone Labs, Jerusalem, Israel (APC-002)), norepinephrine transporter (rabbit polyclonal, 1:200, Alomone Labs, Jerusalem, Israel (AMT-002)) and serotonin receptor 2B (rabbit polyclonal, 1:200, Alomone Labs, Jerusalem, Israel (ASR-035)) were diluted in PBS and incubated with the slides at 4 °C. As a negative control, PBS was used instead of the primary antibodies. After washing, the sections were incubated with biotin secondary antibody for 30 min, and then washed again. The sections were then treated with the Avidin-Biotin (AB) enzyme reagent for 30 min and were washed with the peroxidase substrate, diaminobenzidine (DAB), for 5 min. To stop the DAB reaction, the slides were washed with deionized H_2_O for 5 min. The sections were contrast stained with Gill haematoxylin and were washed several times with deionized H_2_O. Finally, the tissue sections were dehydrated with increasing alcohol series and passed through xylene before covering with Entellan mounting medium and cover slipped. Images were obtained with a DP71 digital camera under the Olympus BX51 model light microscope and the tissue sections were evaluated in terms of immunoreactivity differences with the Image-J software. The interpretation of the data was evaluated depending on the intensity of the staining compared to the control. A significant change in staining intensity in a region was accepted as an indicator of the activity of the related region in terms of the relevant neurotransmitter content. 

### 2.3. Statistical Analysis

The SPSS program (SPSS for Windows, SPSS Inc., Chicago, IL, USA, version 24.0) was used to perform statistical analysis on the behavioural and histology results. Data are given as mean values ± standard error of the mean (SEM). An inter-group comparison of the results was analysed with the one-way ANOVA test, while the independent sample T-test was used for binary comparisons of the results. Differences were considered significant at * *p*< 0.05, ** *p*< 0.01 and *** *p*< 0.001.

## 3. Results

### 3.1. Racine-Scoring of PTZ-Kindling Model in Animals

**Figure 2:**
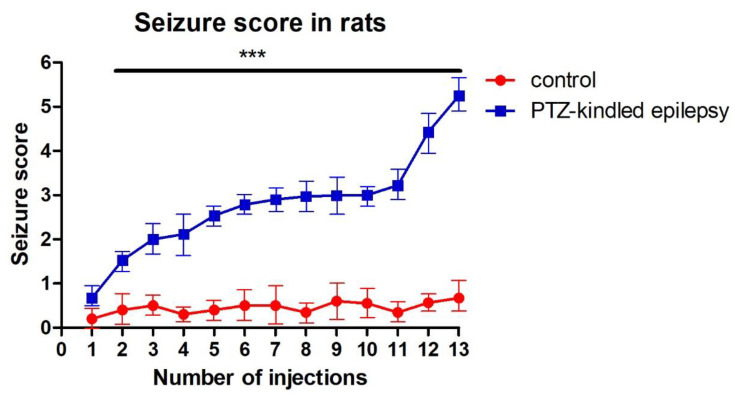
Racine score exhibited by sham control and PTZ-kindled epilepsy rats. Values are presented as the mean ± SEM, *n* = 10 for each group, *** *p* < 0.001.

**Figure 3:**
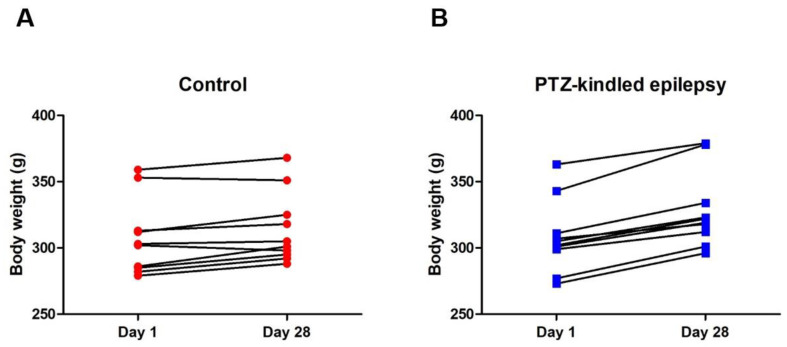
Body weight changes in control (**A**) and PTZ-kindled epilepsy (**B**) groups between day 1 and day 28 of treatment, *n* = 10 for each group, *p* > 0.05.

After the 13th PTZ injection, all Group 2 rats (100%) displayed chronic epilepsy-like behaviour with generalized tonic-clonic seizures (Score 5) (Figure 2, *** *p* < 0.001). The mean seizure latency of all injections in Group 2 was 5.31 ± 0.58, and their mean seizure duration was 308 ± 95 s. No seizure activity was observed in the control animals (Group 1). There were also no observable significant differences in body weight either in the control (307.41 ± 8.98 vs. 314.11 ± 8.47, *p* > 0.05, Figure 3A) or PTZ-kindled epilepsy groups (308.12 ± 8.58 vs. 328.24 ± 9.06, *p* > 0.05, Figure 3B). 

### 3.2. Gross Histopathology Findings

**Figure 4:**
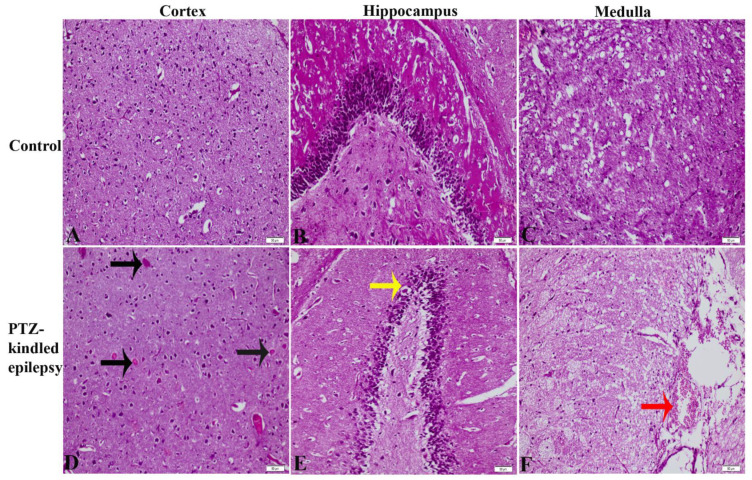
Haematoxylin and Eosin (H&E) staining images of the brain (cortex, hippocampus, and medulla) sections stained with purple in control (**A**–**C**, respectively) and PTZ-kindled epilepsy (**D**–**F**, respectively) groups. The black, yellow, and red arrows denote necrotic cells, cell degeneration, and haemorrhagic areas, respectively. Magnification of ×200. Scale bar: 50 μm.

The H&E-stained tissue images were assessed for gross histopathological changes in the cortex (Figure 4A,D), hippocampus (Figure 4B,E), and medulla (Figure 4C,F). Compared to controls, the PTZ-kindled animals (Group 2) showed increased observations of necrotic cortical cells, neuronal degeneration in the hippocampal cells, and haemorrhagic areas in the medulla oblongata. 

### 3.3. Immunohistochemistry Findings

#### 3.3.1. Upregulation of the Muscarinic ACh Receptor M2

**Figure 5:**
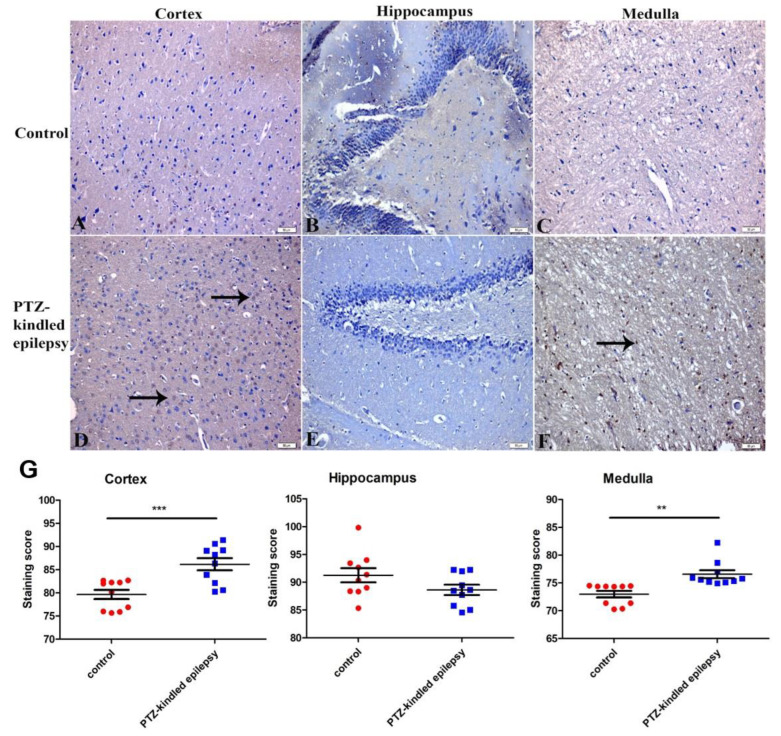
Representative images of M2 receptors within the cortex, hippocampus, and medulla in control (**A**–**C**, respectively) and PTZ-kindled epilepsy of rat model (**D**–**F**, respectively). Figure (**G**) represents the M2 ACh receptor’s immunoreactivity in the cortex, medulla, and hippocampus regions. Values are presented as the mean ± SEM, *n* = 10, ** p < 0.01 and *** p < 0.001. The black arrow indicates the increased immunoreactive cells. Pictures were taken at a magnification of ×200. Scale bar: 50 µm.

Compared to the control group, the PTZ-kindled rats demonstrated significant upregulation of the M2 receptor immunoreactivity in the cortex and the medulla (Figure 5A,C,D,F, respectively). There were no observable/significant intergroup immunoreactivity changes of the M2 receptor in the hippocampus. The PTZ-kindled animals showed an increased M2 Ach receptor immunoreactivity: 86.17 ± 1.32 vs. 79.64 ± 0.99 in the cortex (*** *p* < 0.001) and 76.56 ± 0.73 vs. 72.97 ± 0.59 in the medulla oblongata (** *p* < 0.01) (Figure 5G), when compared to the respective area in the control group. The M2 ACh receptor immunoreactivity in the PTZ-kindled hippocampus showed no significant difference with the control group (88.62 ± 0.92 vs. 91.26 ± 1.27, *p* > 0.05, Figure 3E and Figure 5B).

#### 3.3.2. Upregulation of the Serotonin Receptor 2B (5-HT2B)

**Figure 6:**
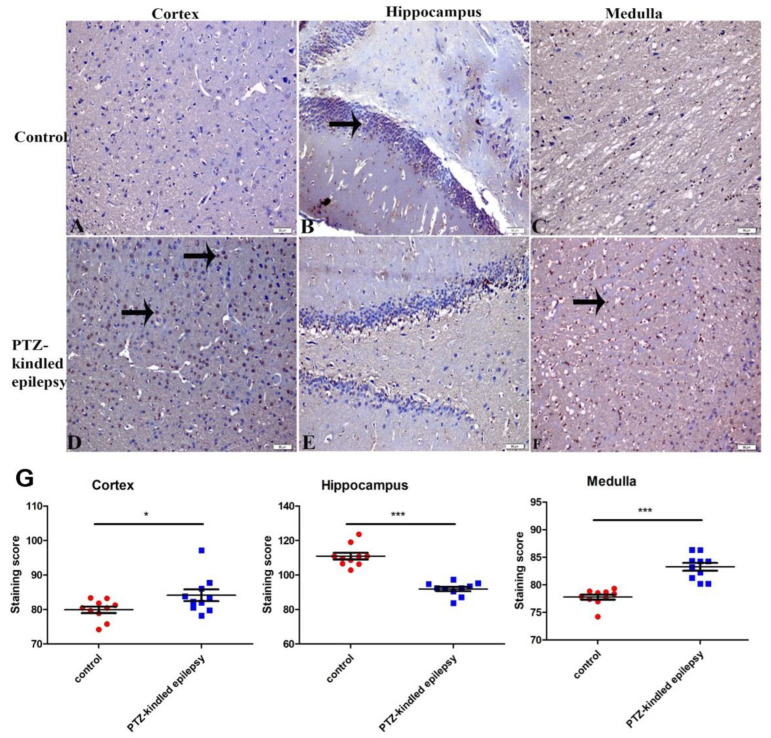
Representative images of immunoreactivity for serotonin receptors within the cortex, hippocampus, and medulla in control (**A**–**C**, respectively) and PTZ-kindled (**D**–**F**, respectively). Figure (**G**) represents the immunoreactivity of the serotonin receptor 2B in several regions (cortex, medulla, and hippocampus). Values are presented as the mean ± SEM, *n* = 10, * p < 0.05, and *** *p* < 0.001. The black arrow indicates the immunoreactive cells. Pictures were taken at a magnification of ×200. Scale bar: 50 µm.

Upregulation of the 5-HT2B immunoreactivity was observed within the cortex, hippocampus, and medulla of the PTZ-kindled rats when compared to the control animals (Figure 6A–F). Compared to the control group, the serotonin receptor 2B immunoreactivity in the PTZ-kindled group was found to be increased in the cortex (84.11 ± 1.70 vs. 79.91 ± 0.94, * *p* < 0.05) and in the medulla (83.26 ± 0.71 vs. 77.78 ± 0.46, *** *p* < 0.001) (Figure 6G). However, the 5-HT2B immunoreactivity was found to be decreased in the hippocampus (91.87 ± 1.26 vs. 110.90 ± 1.94, *** *p* < 0.001) of the PTZ-kindled group when compared to the control group (Figure 6G). 

#### 3.3.3. Increased Immunoreactivity of Norepinephrine Transporter

**Figure 7:**
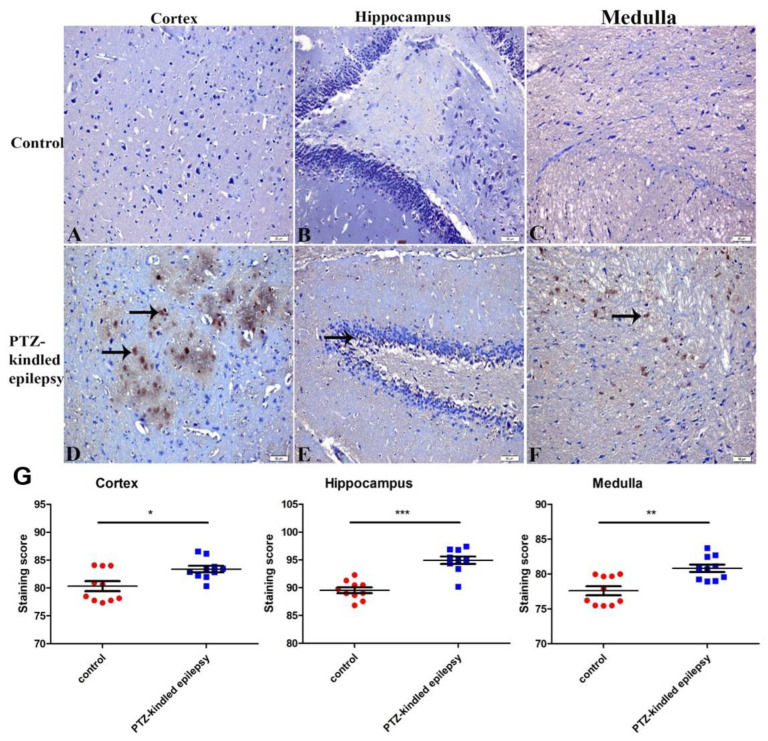
Representative images of immunoreactivity for NE transporter within the cortex, hippocampus, and medulla in control (**A**–**C**, respectively) and PTZ-kindled (**D**–**F**, respectively). Figure (**G**) represents the immunoreactivity of the NE transporter in several regions (cortex, medulla, and hippocampus). Values are presented as the mean ± SEM, *n* = 10, * *p* < 0.05, ** *p* < 0.01 and *** *p* < 0.001. The black arrow indicates the increased immunoreactive cells. Pictures were taken at a magnification of ×200. Scale bar: 50 µm.

The upregulation of NE transporters was witnessed in all three PTZ-kindled brain regions, the cortex, hippocampus and medulla, when compared to the control group (Figure 7A–F). Similarly, the PTZ-kindled rats showed significantly increased immunoreactivity in the NE transporter in the cortex (83.39 ± 0.59 vs. 80.32 ± 0.89, * *p* < 0.05), hippocampus (94.92 ± 0.66 vs. 89.53 ± 0.53, *** *p* < 0.001), and in the medulla (80.83 ± 0.54 vs. 77.59 ± 0.65, ** *p* < 0.01), when compared to the respective brain regions in the control group (Figure 7G). 

#### 3.3.4. Ach-Activated Kir3.1 Channel and ATP-Dependent Kir6.2 Channel 

**Figure 8:**
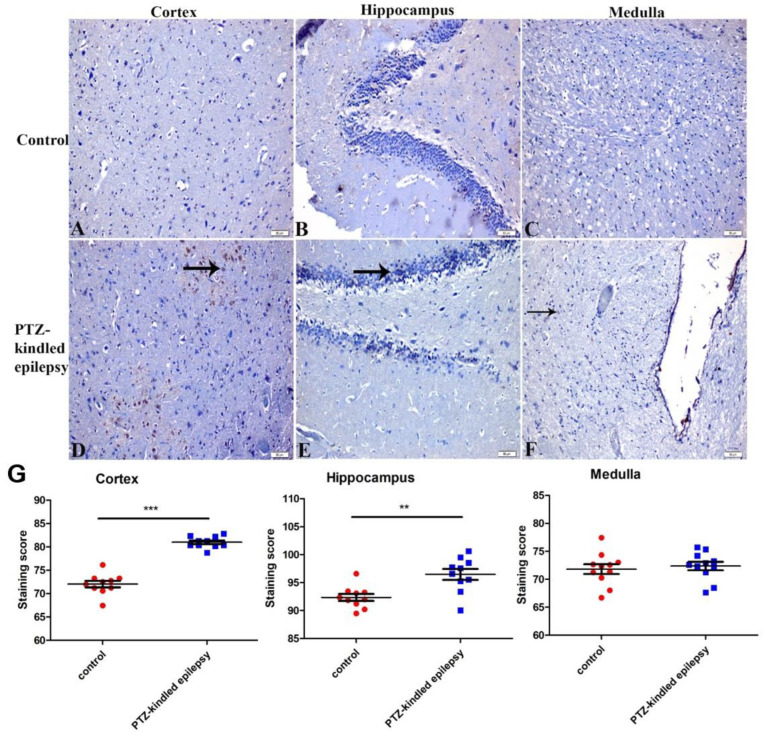
Representative images of immunoreactivity for Kir3.1 channel within the cortex, hippocampus, and medulla in control (**A**–**C**) and PTZ-kindled epilepsy of rat model (**D**–**F**), respectively. Figure (**G**) represents the immunoreactivity of the Kir3.1 channel in several regions (cortex, medulla, and hippocampus). Values are presented as the mean ± SEM, *n* = 10, ** *p* < 0.01 and *** *p* < 0.001. The black arrow indicates the changed immunoreactive cells. Pictures were taken at a magnification of ×200. Scale bar: 50 µm.

**Figure 9:**
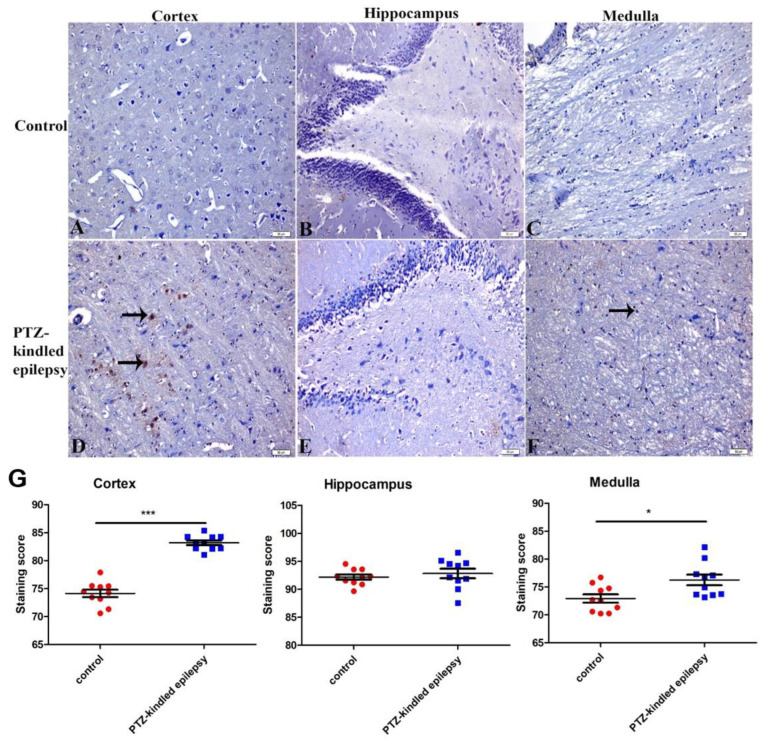
Representative images of immunoreactivity for Kir6.2 channel within the cortex, hippocampus, and medulla in control (**A**–**C**) and PTZ-kindled epilepsy of rat model (**D**–**F**), respectively. Figure (**G**) represents the immunoreactivity of the Kir6.2 channel in several regions (cortex, medulla, and hippocampus). Values are presented as the mean ± SEM, *n* = 10, * *p* < 0.05 and *** *p* < 0.001. The black arrow indicates the changed immunoreactive cells. Pictures were taken at a magnification of ×200. Scale bar: 50 µm.

Immunohistological staining showed an increased immunoreactivity of Kir3.1 channels in the cortex and hippocampus (Figure 8A–F), while an increased immunoreactivity of the Kir6.2 channel was observed in the cortex and medulla (Figure 9A–F) of the PTZ-kindled animals when compared to the control rats. 

In the PTZ-kindled group, an increased Kir3.1 immunoreactivity was observed in the cortex (80.98 ± 0.39 vs. 720.4 ± 0.71, *** *p* < 0.001) and hippocampus (96.47 ± 0.98 vs. 92.35 ± 0.62, ** *p* < 0.01) when compared to the control animals, but there were no significant Kir3.1 immunoreactivity differences observed in the medulla between the two groups (72.36 ± 0.76 vs. 71.81 ± 0.88, *p* > 0.05, Figure 8G). 

The PTZ-kindled group also had an increased immunoreactivity of Kir6.2 in the cortex (83.20 ± 0.42 vs. 74.14 ± 0.68, *** *p* < 0.001) and the medulla (76.26 ± 0.97 vs. 72.93 ± 0.75, * *p* < 0.05), respectively, when compared with the control group (Figure 9G). No significant differences in Kir6.2 immunoreactivity were observed in the hippocampus of the PTZ-kindled animals when compared to the control group (92.82 ± 0.86 vs. 92.18 ± 0.46, *p* > 0.05).

## 4. Discussion

The findings from our study demonstrated that chronic PTZ-kindling induces location-specific alterations in the immunoreactivity of ion channels as well as of neurotransmitter receptors and transporters, which may play a role in ictogenesis. We found significantly increased immunoreactivity of the NE transporter, the M2 and 5-HT2B receptors as well as the Kir3.1 and Kir6.2 ion channels in the cortex of the PTZ-kindled rats when compared to the sham controls. As for the medulla, only the Kir3.1 ion channel did not show any significant upregulation when compared to sham controls. However, in the hippocampus, only the NE transporters and Kir3.1 channels showed increased immunoreactivity, but the 5-HT2B receptors showed a decrease in immunoreactivity, while the M2 receptors and Kir6.2 channels showed no significant changes in immunoreactivity when compared to controls. This supports the notion that different brain areas may have different pathological processes/roles in epilepsy and thus should be taken into consideration in future pathological studies. We believe that these kindling-induced changes in immunoreactivity may contribute to ictogenesis and even epileptogenesis, or may cause compensatory effects that will decrease excitability and result in epiphenomena. However, these speculations will require future studies to effectively assess the potential pathogenic/ictogenesis/epileptogenesis role of each of these immunoreactivity changes in a more counterintuitive way.

Neurotransmitters may excite, inhibit or modulate neuronal activity through receptors/transporters and ion channels, thereby potentially playing a crucial role in altering seizure threshold/activity and epileptogenesis. For example, mutations in the *CHRNA4* gene, which encodes for the nicotinic ACh receptor subunit, may cause the development of nocturnal frontal lobe epilepsy [27], while pilocarpine, a nonselective muscarinic receptor agonist, may initiate seizures and status epilepticus in experimental animals [28]. Similarly, the 5-HT receptor has been shown to alter seizure thresholds in PTZ, kainic acid, and penicillin animal epilepsy models. The 5-HT1A receptor agonists have been observed to prolong the seizure latency and decrease the frequency of PTZ-induced tonic-clonic convulsions as well as of kainic acid-induced status epilepticus [29]. As with the 5-HT1A receptor, the 5-HT2 receptor agonists have also been suggested to reduce epileptiform activity in a penicillin epilepsy model, which was found to be counteracted (increased epileptiform activity) with a 5-HT2 receptor antagonist treatment, methysergide [30]. Hence, in our study, the increased expression but decreased immunoreactivity (inhibition) of the 5-HT2B receptors in the hippocampus may contribute to the epileptogenesis process, while the increased immunoreactivity of the receptor in the cortex and medulla may indicate a more neuroprotective role of serotonin against seizures. Seizure-related increases in serum 5-HT levels are associated with a lower incidence of respiratory dysfunction (role of medulla), which may provide a protective effect against sudden unexpected death in epilepsy (SUDEP) [31]. However, in surgical tissues from TLE patients, low hippocampal serotonin levels were significantly correlated with a history of generalized tonic-clonic seizures [32]. Thus, our results support that the seizure threshold may be reduced due to decreased hippocampal 5-HT2B receptor immunoreactivity, but further behavioural/functional outcomes should be performed to determine if the increases in the cortex and medulla may provide protection against the seizure kindling as well. 

Interestingly, transgenic mice lacking the 5-HT1A or 5-HT2C receptors tend to develop epilepsy, with an increased seizure frequency that was associated with a decrease in 5-HT and NE levels [33]. Decreased NE levels are related to the increased expression of the norepinephrine transporter (NET), an inverse relationship. The NET removes NE from the synapse of noradrenergic neurons and also plays a role in dopamine reuptake in the hippocampus and cortex [34]. Norepinephrine has anti-seizure and antiepileptic effects in animal models [35], thus a decrease in NE may result in ictogenesis and epileptogenesis [36]. In our PTZ-kindled model, the NET immunoreactivity was upregulated in the hippocampus, medulla, and cortex, suggesting a possible decrease in NE levels which therefore may propagate epileptogenesis. Since the neurotransmitter levels were not investigated in this study, whether the epileptogenesis pathway was initiated by either the decrease in NE or the decrease in dopamine levels remains to be determined. 

As for the Kir ion channels, Kir6.2 channel immunoreactivity has been shown to be increased after seizures in a cellular epilepsy model established by culturing hippocampal neurons in a magnesium-free medium [37]. Similarly, the seizure threshold was seen to be decreased in a mice model with genetically down-regulated Kir6.2 channels [38]. On the other hand, Kir3 channels may exhibit a duality in epileptogenesis; pro-convulsant or anti-convulsant effect. Studies show that inhibiting Kir3 channels results in seizure development and activating Kir3 channels may prevent epileptogenesis, but kindled seizures may increase the immunoreactivity of Kir3 channels as a compensatory mechanism towards neuroprotection against excessive stimulation [39]. Thus, the increased immunoreactivity of Kir3.1 channels in our study may suggest a neuroprotective role against the kindled seizures, but further studies are needed to determine if the increased immunoreactivity propagates epileptogenesis thereafter. The opposite may be true for the increased immunoreactivity of the Kir6.2 channels, as they may result in increased seizure activity instead.

Epilepsy/seizures may affect the autonomic nervous system, resulting in cardiac and respiratory dysfunctions, a precursor towards SUDEP [40]. Studies have shown that the 5-HT transport may be impaired in the ventrolateral medulla (VLM) and medullar raphe (MR) regions, which are involved in respiratory control in SUDEP conditions [41]. Moreover, lesions in 5-HT neurons projecting to respiratory nuclei may impair autonomic function by suppressing respiration after seizures [42]. Using functional imaging, receptor immunoreactivity changes were also found in the connections between the autonomic regulatory cortex regions and the brainstem [43]. This, taken together with the loss/lesion of serotonergic neurons and specialized glial cells in VLM and medullary raphe (MR), may cause impairment in the responsiveness of respiratory neurons during or after seizures [44], thereby contributing to the SUDEP risk. In addition, the opening of the Kir6.2 channels in the brainstem neurons may synchronize with the respiratory firing rhythm [45]. In our study, we found increased expression of the medullar Kir6.2 channels after PTZ-kindling, suggesting the possible desynchronization (rapid firing) of the respiratory system, which unfortunately was not investigated but may be warranted in future studies. Besides that, increased medullary M2 receptor immunoreactivity, similar to our study, may also contribute to the impairment in cardiorespiratory regulation, especially when in combination with Kir3.1 upregulation [26]. Future studies should perform ion-current measurements of the Kir channels in association with the immunoreactivity changes of M2 receptors in the medulla in order to better understand the underlying mechanisms relating epilepsy to cardiorespiratory dysfunctions. 

This study also observed gross pathological changes; necrosis in the cortex, cellular degeneration in the hippocampus and haemorrhage in the medulla, in the PTZ-kindled rats compared to the controls. Seizures may increase heart rate, blood pressure, neuronal metabolism, and cerebral blood flow, which may result in brain damage due to adenosine triphosphate (ATP) depletion and lactate accumulation caused by hypermetabolic neural necrosis [46]. Hippocampal damage and reduced glucose metabolism have also been reported previously after PTZ-induced seizures, where they were associated with increased oxidative stress found in the hippocampus as well as in the cerebral cortex [47]. In the PTZ-kindling model, severe necrosis and degeneration with vacuolization were also observed in the hippocampal neurons in a past study [48]. On the contrary, this pathology in our model may have also been observed due to the final overdose (50 mg/kg, i.p.) of PTZ in the animals, resulting in severe neurotoxic pathology in the brain. 

## 5. Conclusions

In conclusion, chronic/long-term PTZ-kindling was associated with increased immunoreactivity of neurotransmitter receptors/transporters and ion channels, which may govern the neurotransmitter levels such as ACh, serotonin, and NE in the cortex, hippocampus, and medulla. Although these changes may have resulted from seizure kindling, they may also contribute to the molecular pathogenesis of epilepsy in the long run. Our findings suggest that alternative therapeutic approaches may target these neurotransmitter receptors/transporters, as well as the alterations in Kir channel function, to treat epilepsy, especially drug-resistant epilepsy, and prevent epilepsy related autonomic comorbidities such as SUDEP, thereby improving the future lives of epileptic patients. 

## Data Availability

Not applicable.
